# Newly Diagnosed Acute Promyelocytic Leukemia

**DOI:** 10.4084/MJHID.2011.064

**Published:** 2011-12-20

**Authors:** Giuseppe Avvisati

**Affiliations:** Unità Operativa Complessa di Ematologia, Trapianto di cellule staminali, Medicina trasfusionale e Terapia cellulare. Università Campus Bio-Medico, Roma, Italy

## Abstract

Acute promyelocytic leukemia (APL) represents a medical emergency with a high rate of early mortality. As a consequence, as soon as the diagnosis is suspected based upon cytologic criteria, it is necessary to start all- trans retinoic acid (ATRA) treatment without delay. For patients with newly diagnosed APL, induction therapy with ATRA plus anthracycline based chemotherapy is recommended. At present the combination of arsenic trioxide plus ATRA should be considered for patients who are not candidates for anthracycline-based therapy. For pediatric and adult patients with APL aged < 60 years who achieve a CR with induction, I recommend 3 intensive courses of consolidation chemotherapy associated to ATRA, targeted on the basis of the risk group at diagnosis. In patients treated with a very intensive consolidation chemotherapy maintenance treatment can be omitted. However If a maintenance treatment has to be adopted I suggest the use of intermittent ATRA for 15 days every 3 months for a period of 2 years, rather than ATRA associated to chemotherapy. Moreover, taking into account the medical literature, a reduced dosage of ATRA ( 25 mg/m^2^) in pediatric patients and a consolidation chemotherapy of reduced intensity in elderly patients is recommended. Furthermore, in order to maximize survival, careful attention should be reserved to the coagulopathy and to the appearance of the differentiation syndrome. Finally, PCR for the PML/RARA fusion gene on a bone marrow specimen every three months for two years, and then every six months for additional three years are needed during the follow-up.

## Introduction

Acute promyelocytic leukemia (APL) represents a biological and clinical distinct variant of acute myeloid leukemia (AML) characterized by a typical blast cell morphology, presence of a severe coagulopathy, high sensitivity to induction chemotherapy with anthracycline drugs and by the presence of the specific chromosome translocation t (15;17) in the leukemic blasts.^1^–^4^ This translocation involves the retinoic acid receptor-alfa (RARA) gene on chromosome 17 and the PML (for promyelocytic) gene on chromosome 15 giving origin to the chimeric genes PML/RARA and RARA/PML.^5^–^7^ In the older French-American-British (FAB) classification system APL was classified as AML-M3 while, in the WHO classification system, is currently classified as acute promyelocytic leukemia with t(15;17)(q22;q12);PML-RARA.^8^

Without treatment, APL is the most malignant form of AML with a median survival of less than one month^9^–^11^ and many patients die before reaching an experienced hematologist. However, modern therapy has dramatically changed its prognosis and APL is now associated with the highest proportion of AML patients who are cured of their disease. *In this article I will give my personal opinions about the modern treatment of newly diagnosed APL, a treatment distinct from that of other types of AML*.^12^–^13^

## What to do in case of suspected APL?

This is the first question that I would like to address because, as in the other AML, all patients with suspected APL should undergo several studies to evaluate disease extent and comorbidities; however, these studies should not interfere with the rapid initiation of ATRA-based therapy. In fact, APL represents a medical emergency with a high rate of early mortality, due mainly to hemorrhagic complications from a characteristic coagulopathy. Therefore, **the first golden rule** that has to be followed as soon as the diagnosis of APL is suspected based upon cytologic criteria **is**: *to immediately start treatment with all-trans retinoic acid (ATRA) without delay, even before definitive (cyto)genetic confirmation of the diagnosis has been made*.^14^ If the diagnosis is not confirmed, ATRA can always be discontinued and treatment changed to that used for other types of AML.

## What is the best induction therapy?

In the following sections I will describe some of the multiple trials that have investigated the optimal means of combining ATRA and chemotherapy during induction.

### ATRA alone

ATRA was firstly utilized as single agent to treat APL by a Chinese group from Shangai in 1988.^15^ However, it was soon evident that remissions induced by ATRA therapy alone were short-lived with a median duration of less than 6 months.

### ATRA plus Chemotherapy

Very soon, after the experience of the Shangai group, ATRA was combined with anthracycline-based chemotherapies. The concurrent administration of ATRA plus cytotoxic chemotherapies produced a complete remission (CR) rate in more than 80 percent of patients of all ages with newly diagnosed APL^16^–^20^ and almost all treatment failures were due to early mortality,^21^ whereas primary resistance to the combination of ATRA and chemotherapy was unusual except for patients with the rare t(11;17) who must generally be treated as the other AML.

The concurrent administration of ATRA plus chemotherapy was supported by randomized trials demonstrating that patients who received both ATRA and chemotherapy had superior rates of CR and disease-free survival when compared with patients who received chemotherapy alone.^16^–^17^ Moreover, the duration of coagulopathy was reduced when ATRA was added to induction chemotherapy.^16^,^22^–^23^ Furthermore, the addition of chemotherapy to ATRA helped to control hyperleukocytosis that occurred more frequently when ATRA was used alone. Therefore, the combination of ATRA plus chemotherapy limits complications that may occur when either agent is used alone.

### Timing of Chemotherapy

Several trials have demonstrated better outcomes when chemotherapy is administered with ATRA during induction (simultaneous administration) rather than postponing chemotherapy until a CR is achieved with ATRA (sequential administration). The largest of these trials was a prospective randomized trial comparing sequential and simultaneous administration of ATRA and chemotherapy in more than 400 patients with newly diagnosed APL.^16^ While the rates of CR were similar in the two treatment groups, patients who received simultaneous therapy had significantly lower rates of relapse at two years (6 versus 16 percent) and a non significant lower rates of differentiation syndrome (11 versus 20 percent) when compared with those who received sequential therapy. After a median follow-up of 10 years, this lower relapse rate persisted, but lost its statistical significance (13 versus 22 percent, respectively).^24^

### Choice of Chemotherapy

For many years anthracyclines have been the mainstays of induction therapy for APL; the observation of an exquisite sensitivity of APL to daunorubicin, originally reported by Bernard et al in 1973,^25^ was confirmed and extended to other anthracyclines in the 1980s.^26^–^29^ Unlike other AML subtypes, these agents were able to produce long-lasting remissions when used as induction monotherapy for APL. A possible explanation of such high sensitivity of APL to anthracycline drugs maybe the low expression of the multidrug-resistance related protein (MDR-1) on the membrane of leukemic cells.^30^–^33^ Therefore, because ATRA therapy alone induced short-lived remissions, anthracycline-based regimens associated to ATRA have been administered to patients with APL. The best results have been obtained when ATRA was combined with daunorubicin and cytarabine 16,34–37 or with idarubicin alone.^20^,^38^–^40^ These trials have reported a CR rate ranging from 80% to 95%. Moreover, a prospective, randomized trial that investigated whether cytarabine could be eliminated from this regimen found that patients who received cytarabine had similar CR rates but fewer relapses and improved survival, when compared with patients who did not receive cytarabine.^37^,^41^ However, the PETHEMA group has produced excellent outcomes with successive induction and consolidation cycles using idarubicin and mitoxantrone without the use of cytarabine.^19^ A retrospective analysis of patients treated with AIDA induction reported a CR rate of 91 percent.^42^ The most common causes of early mortality were hemorrhage (5 %) and infection (2.3 %) with lethal bleeding occurring most frequently in the first week of therapy while deaths due to infection were spread throughout the course of induction treatment.

## How I will induce hematological CR in newly diagnosed APL?

Taking into account all the previous observations, *for patients with newly diagnosed APL, I recommend induction therapy with ATRA plus anthracycline-based chemotherapy rather than treatment with either ATRA or chemotherapy alone. In Italy*, *the combination of ATRA with the anthracycline Idarubicine (AIDA)*, *without the use of cytarabine, is the preferred induction treatment of newly diagnosed APL*. This induction treatment was firstly proposed by the GIMEMA group 43 and consist of daily ATRA (45 mg/m^2^/day orally divided into two doses), starting on day zero, and four days of idarubicine (12 mg/m^2^ days 1,3,5,7). Children and adolescents (< 20 years) should receive a reduced dose of ATRA (25 mg/m^2^ per day) in combination with chemotherapy.^40^ ATRA is continued throughout the period of pancytopenia until a CR is obtained. This regimen requires transfusion support and antibiotics as needed. Daily laboratory testing generally includes a complete blood count, renal and liver functions, glucose and electrolytes. Calcium, phosphorus, and uric acid levels should be monitored until normal. Coagulation parameters, should be monitored closely. Common non-hematologic side effects include stomatitis, reversible alopecia, nausea and vomiting. Beside minor drug toxicities due to ATRA such as: headache; nasal stuffiness, dry red skin, chapped lips, transient elevations in serum aminotransferases and bilirubin, and hypertriglyceridemia there are two serious and specific complications that can result from ATRA treatment: differentiation syndrome and hyperleukocytosis. These complications are discussed in more detail below.

## Consolidation treatment

Generally, with induction therapy, 90 percent of patients with newly diagnosed APL achieve a hematological complete remission (CR). However, to avoid a relapse in all these patients, additional cytotoxic consolidation therapy is needed. The aim of consolidation therapy is to eliminate leukemia cells that survived induction therapy and were not detectable by conventional tests. Therefore, by consolidation treatment, patients with morphologic and cytogenetic CR after induction will be converted into a more durable molecular remission and eventually a cure. Several studies have shown a highly significant correlation between patients’ molecular status detected at the end of consolidation and subsequent outcome.^44^,^45^ This is the reason why *current guidelines have established that molecular remission must be a therapeutic objective in APL*.^46^

### How should APL be consolidated?

A consolidation treatment with 2–3 intensive cycles of an anthracycline (daunorubicin or idarubicin) based chemotherapy should be adopted as standard in this phase of treatment because it is able to produce a PCR-negativity in about 99% of APL.

### Can post-remission therapy be risk-adapted?

Patients with APL can be risk-stratified for relapse into three groups based upon WBC and platelet counts at diagnosis:^47^

- Low-risk disease (WBC count <10,000/μL and platelets >40,000/μL).
- Intermediate-risk disease (WBC count <10,000/μL but platelets <40,000/μL).
- High-risk disease (WBC count >10,000/μL and platelets <40,000/μL).

Using current regimens, there is little difference in outcomes between the low-risk and intermediate-risk groups.

A PETHEMA prospective trial examined the use of risk-adapted consolidation therapy in APL patients in first CR after induction with AIDA regimen.^48^ The results revealed that patients with low- or intermediate-risk disease, who received less intensive consolidation, when compared with historical matched controls, had shorter durations of neutropenia and thrombocytopenia while patients with high-risk disease who had cytarabine added to consolidation demonstrated a significantly lower rate of relapse at three years (11 versus 26 percent).

A second prospective trial of risk-adapted therapy by the GIMEMA group 39 assigned patients with low-or intermediate-risk disease to consolidation with three anthracycline-based courses, while patients with high-risk disease had cytarabine-based consolidation. In particular, patients with low-/intermediate-risk received the same 3 consolidation courses as in the AIDA-0493 40 but with omission of cytarabine from courses 1 and 3 and omission of etoposide from course 2; patients in the high-risk group received the identical 3 cycles as in the AIDA-0493.^40^ In addition, distinct from the AIDA-0493, oral ATRA at 45 mg/m^2^ per day for 15 days was added in the AIDA-2000 at the start of each consolidation course for all risk groups. When compared with historical matched controls, patients with low- and intermediate-risk disease had significantly improved disease-free survival rates at six years (86 versus 77 percent) and a lower cumulative incidence of relapse (11 versus 20 percent), but similar rates of overall survival (89 versus 85 percent). Patients with high-risk disease had superior overall survival at six years (83 versus 61 percent), disease-free survival (85 versus 50 percent), and a lower cumulative incidence of relapse (9 versus 50 percent).

These two studies indicate that *risk adapted therapy is feasible and save even though a longer follow-up and more studies of these approaches are needed before risk-adapted therapy can be widely implemented*.

## Evaluation of the response after consolidation

The aim of APL treatment is the achievement of a molecular complete remission (CRm) at the end of the consolidation phase. CRm is defined by the absence of the PML-RARA fusion transcript using RT-PCR methods. Therefore, after the completion of consolidation, the response to treatment must be evaluated with a bone marrow aspirate tested for the PML-RARA fusion transcript using reverse transcription polymerase chain reaction (RT-PCR), with a sensitivity threshold of at least 10^−4^.^46^ Because a CRm takes time to achieve, a premature evaluation of CRm by RT-PCR should not be attempted immediately after completion of induction treatment to avoid a misleading interpretation of response failure. Patients who achieve CRm after completion of consolidation should proceed directly to maintenance therapy. Patients who result positive for the PML-RARA fusion gene at the end of the planned consolidation sequence should have a second bone marrow aspirate with RT-PCR testing repeated in four weeks. If this second test is negative, the patient may proceed to maintenance therapy. If the second RT-PCR is still positive, the patient should proceed to treatment for resistant disease.

## What about the use of maintenance treatment in Molecularly negative APL after Consolidation?

The first prospective randomized trials of ATRA plus chemotherapy demonstrated that, when compared with observation, patients assigned to ATRA maintenance had superior rates of disease-free survival at five years and a lower 10-year cumulative incidence of relapse.^24^,^35^ The main side effects of maintenance therapy are cytopenias and liver enzyme elevation 24 easily managed in most patients with dose adjustments. However, two recent randomized trials dealing with maintenance therapy after the achievement of a CRm have demonstrated that maintenance therapy in APL may not be necessary.^40^,^49^ In both these studies all patients were in CRm following consolidation and have received a more intensive consolidation therapies than those utilized in early studies. In particular, the JALSG study involving 175 patients in CRm following induction and three cycles of consolidation chemotherapy, the addition of six courses of intensive maintenance chemotherapy, as compared with observation only, unexpectedly conferred significantly poorer six-year disease-free survival (63 versus 80 percent) and overall survival (86 versus 99 percent).^49^ Moreover, in the GIMEMAstudy including 586 patients with newly diagnosed APL in CRm after induction with ATRA and idarubicin followed by three cycles of intensive consolidation therapy, maintenance did not appear to improve disease-free survival.^40^ Until additional data are available, *my personal opinion about the use of maintenance treatment is to avoid maintenance treatment in APL, provided that patients are in CRm and have received an intensive 3 courses consolidation phase, or to use ATRA 45 mg/m^2^PO daily on an intermittent schedule (15 days every three months) for 2 years*.

### Monitoring response after consolidation

Once a CRm is achieved patients must be followed, using bone marrow aspirate samples, with RT-PCR for the PML-RARA fusion transcript every 3 months during the first 2–3 years and then every 6 months for additional 2 years in order to monitor for molecular relapse. If CRm is lost, the patient undergoes bone marrow evaluation for confirmation within two weeks and proceeds to therapy for relapsed disease.

## Complications that are specific to patients with APL

### Control of coagulopathy

APL is characterized by the presence of a severe coagulopathy involving the coagulation and fibrinolityc systems with the participations of some proteolytic enzyme and some cytokines.^22^ Therefore, treatment of the coagulopathy associated with APL may be difficult and should be managed expectantly. Coagulation parameters (fibrinogen, FDP/XDP, PT, aPTT, and platelet counts) should be monitored closely. Our approach to the control of coagulopathy is mainly based upon our own clinical experience and observations and may be summarized as follows:

- *Transfusions of platelets or fresh frozen plasma are traditionally used to maintain the platelet count above 20,000 to 30,000/*μ*L and the plasma fibrinogen concentration above 150 mg/dL*.
- Heparin must not be used for prophylaxis in this setting.
- In case of life-threatening bleeding, inhibitors of fibrinolysis should be considered.
- Invasive procedures such as central venous catheterization, lumbar puncture, and bronchoscopy should be avoided before and during induction remission.

### Differentiation syndrome

This syndrome, previously named *"retinoid acid syndrome"*, occurs in 10–25% of APL patients within 2 to 21 days after initiation of treatment and is seen more frequently in patients with a high white blood cell count at diagnosis.^50^–^51^ It is characterized by fever, peripheral edema, pulmonary infiltrates, hypoxemia, respiratory distress, hypotension, renal and hepatic dysfunction, and serositis resulting in pleural and pericardial effusions. The symptoms of fever, hypotension, dyspnea, and pulmonary infiltrates can mimic sepsis. Sometimes, the syndrome is accompanied by hyperleukocytosis. *Early recognition and aggressive management with dexamethasone therapy (10 mg IV every 12 hours for three or more days) has been effective in most patients*. After respiratory distress is established temporary cessation of ATRA or ATO, leukapheresis, or prompt institution of cytotoxic chemotherapy have not been effective. ATRA or ATO can be restarted in most cases once the syndrome has resolved.

### Hyperleukocytosis

The marked increase in WBC count due to the rapid maturation induced by ATRA of a large mass of leukemic cells, may result in leukostasis and management is controversial. However, because the most current remission induction regimens now combine ATRA with cytotoxic chemotherapy, the frequency of hyperleukocytosis has decreased. Despite patients with an elevated WBC count have a similar rate of complete remission compared with those with a normal or decreased white blood cell (WBC) count, however they have a higher rate of relapse and are more likely to develop the differentiation syndrome.^51^–^52^

### Pseudotumor cerebri

Idiopathic intracranial hypertension (IIH), commonly called pseudotumor cerebri, can complicate the treatment of APL with ATRA.^53^ It is more common in children and adolescents treated with ATRA and the incidence in this population decreased with the use of lower dose ATRA (25 mg/m2/day). The diagnosis of IIH is suspected in patients with headache, papilledema, and/or vision loss. Evaluation includes a physical examination including lumbar puncture, cerebral imaging studies (computed tomography or magnetic resonance) and *fundus oculi.* The diagnosis is confirmed in patients with increased intracranial pressure, normal cerebrospinal fluid, and negative cerebral imaging studies. If symptoms persist, therapeutic options include the temporary discontinuation or dose reduction of ATRA, analgesics, and/or the administration of steroids and acetozolamide

## Management of special situations

Some patients with APL such as pregnant women, therapy-related APL, those with genetic variations of APL as well as elderly and pediatric patients require special consideration.

### Pregnant women

Patients diagnosed with APL during pregnancy pose a distinct challenge requiring a team approach with a hematologist, obstetrician, and neonatologist. The treatment approach depends largely upon the trimester of pregnancy during which APL is diagnosed.^54^–^55^

#### First trimester

Both ATRA and ATO are considered to be highly teratogenic and are contraindicated during the first trimester of pregnancy. Therefore, if elective termination of the pregnancy is unacceptable to the patient, the only available treatment option is the administration of chemotherapy alone. If treatment with chemotherapy alone is chosen, daunorubicin may be the preferred anthracycline for pregnant women because there is greater experience with this drug during pregnancy.^54^–^55^ If a remission is achieved and the pregnancy continues normally, ATRA may be added during the second or third trimester.

#### Second or third trimester

Two main options are available for women who are diagnosed with APL in the second or third trimester of pregnancy:

- Remission induction with ATRA alone and chemotherapy postponed until after delivery.
- Simultaneous administration of ATRA plus chemotherapy.

The simultaneous administration of ATRA plus chemotherapy offers the best chance of cure. Vaginal delivery is generally preferred since it is associated with a reduced risk of bleeding. After delivery, breastfeeding is contraindicated while on chemotherapy or ATO.

### Therapy related APL (t-APL)

Patients with t-APL appear to have a similar prognosis as de novo APL and benefit from standard APL therapy.^56^ Therefore, most patients with t-APL can be treated with standard APL therapy. In patients with a history of anthracycline exposure or cardiac impairment that limits their ability to receive further treatment with anthracyclines, alternative regimens, such as ATRA plus ATO may be used.

### Genetic Variations of APL

More than 90% of patients with APL have t(15;17)(q22;q12) translocation resulting in the PML/RARA fusion gene. However, very rarely have been described alternative fusion genes resulting in leukemias classified as "AML with a variant RARA translocation" and some of these conditions are sensitive to ATRA therapy while others are not.

In general, patients with alternative fusion genes ATRA-sensitive are treated with standard ATRA-based therapy, as described above. Patients with variants known to be resistant to ATRA are treated with standard AML induction therapy. The following are the alternative fusion genes identified as ATRA-sensitive:

- NuMA/RARA and t(11;17)
- NPM1/RARA and t(5;17)
- FIP1L1/RARA

Whereas, the following are ATRA-resistant variants:

- STAT5b/RARA and interstitial chromosome 17 deletion
- PLZF/RARA and t(11;17)

In case APL patients have additional cytogenetic abnormalities, as trisomy 8, or particular molecular abnormalities (gene mutations in FLT3) the prognosis is not worsened and they are considered to have the same prognosis as standard APL.

### Elderly APL patients

Because of the possibility of an increased therapy-related toxicity, elderly patients (60 years or older) are usually treated with less intensive regimens..^57^,^58^

### Pediatric APL patients

So far only few studies, have reported therapeutic results using ATRA plus Chemotherapy in pediatric patients. Compared to the disease in adults, APL diagnosed in childhood more frequently presents with hyperleukocytosis. In spite of that, the outcome results are comparable. With the aim of decreasing the risk of pseudotumor cerebri, the dose of ATRA in this setting has generally been reduced to 25 mg/m^2^ without compromising the results.^59^–^61^

## Do I have to use CNS prophylaxis in APL?

Generally in newly diagnosed APL patients the central nervous system (CNS) involvement is very rare if not absent. As a consequence, CNS has been never routinely used. However, recently an increased number of CNS relapses have been reported suggesting a possible association with ATRA use. This correlation, however, was not demonstrated by a large retrospective study of the GIMEMA group carried out in two cohorts of patients included in two successive therapeutic protocols utilizing or not ATRA during the induction treatment.^62^ It is therefore possible that the increased survival observed in APL patients treated with ATRA-based regimens, may account for the apparently higher prevalence of extramedullary relapse, including CNS relapse. As a consequence, while there is a general consensus to avoid CNS prophylaxis for patients without hyperleukocytosis (in whom the risk of CNS relapse is extremely low), *it is my personal opinion that because the majority of CNS relapses occur in patients with hyperleukocytosis, CNS prophylaxis with methotrexate 12 mg (total dose) and 6-methyl prednisolone 40 mg (total dose) is needed for patients in this particular high-risk setting.* Moreover, because lumbar puncture at presentation and during induction is extremely hazardous, *CNS prophylaxis should be performed only after the achievement of CR.*

## When Hemopoietic Stem Cells Transplantation (HSCT) is needed?

For those APL patients who are in the first molecular remission at the end of consolidation, considering the high cure rate obtained using upfront ATRA and chemotherapy, there is no role for HSCT. On the contrary, for the small fraction of patients with persistent minimal residual disease at the end of consolidation, considering the overall poor prognosis of this subset of patients,^63^ ATO and/or Gemtuzumab (GO) followed by HSCT should be considered. Allogeneic HSCT is the recommended choice for patients with an available HLAidentical donor, whereas autologous HSCT is a valid alternative for patients ineligible for allogeneic transplant even though the achievement of PCR-negativity prior to autologous HSCT is a mandatory requisite.

## The role of Arsenic Trioxide (ATO) in newly diagnosed APL

ATO alone or in combination with ATRA, is capable of producing CRm in previously untreated APL.^64^–^69^

### When to use ATO plus ATRA as induction treatment in newly diagnosed APL?

At present, this combination of ATO plus ATRA should be reserved to APL patients who are not candidates for anthracycline-based therapy. However, some studies are evaluating the possibility of avoiding chemotherapy and using this type of combination in low risk (WBC count <10,000/mm^3^ and platelets >40,000/mm^3^) APL patients.

Moreover, the combination of ATO plus ATRA has not been directly compared with ATRA plus anthracycline-based chemotherapy. Therefore, *for most patients with newly diagnosed APL, I prefer ATRA plus chemotherapy as induction treatment mainly because of the greater experience and longer follow-up of patients treated with this combination*. However, *ATO plus ATRA may be an excellent option for patients, such as older adults, who are not able to tolerate anthracycline-based therapy*.^13^

## Conclusions

Acute promyelocytic leukemia (APL) represents a medical emergency with a high rate of early mortality. It is necessary to start ATRA treatment without delay as soon as the diagnosis is suspected based upon cytologic criteria. For patients with newly diagnosed APL, I recommend induction therapy with ATRA plus anthracycline based chemotherapy. At present the combination of arsenic trioxide plus ATRA should be considered for patients who are not candidates for anthracycline-based therapy. For pediatric and adult patients with APL aged < 60 years who achieve a CR with induction, I recommend 3 courses of a consolidation chemotherapy associated to ATRA, targeted on the basis of the risk group at diagnosis. In those patients who achieve CRm after a risk adapted consoldiatation treatment, I do not suggest the use of maintenance therapy. However, if a maintenance treatment has to be adopted I suggest the use of intermittent ATRA for 15 days every 3 months for a period of 2 years. Moreover, I suggest a reduced dosage of ATRA (25 mg/m^2^) in pediatric patients and a consolidation chemotherapy of reduced intensity in elderly patients with the possibility of using the combination of ATO+ATRA in patients who are not candidates to anthracycline-based therapy. Furthermore, careful attention in order to maximize survival should be reserved to the coagulopathy present in APL using transfusions of platelets to maintain the platelet count above 30,000/μL or higher associated to the immediate initiation of treatment with ATRA plus chemotherapy. Careful attention should be reserved also to the appearance of the differentiation syndrome because the early recognition and the immediate treatment with dexamethasone (10 mg IV every 12 hours for three or more days), along with temporary cessation of ATRA is paramount. ATRA can be restarted once the syndrome has resolved. Finally, PCR for the PML/RARA fusion gene on a bone marrow specimen every three months for two years, and then every six months for two years are needed during the follow-up.
